# Estimating Prevalence of Coronary Heart Disease for Small Areas Using Collateral Indicators of Morbidity

**DOI:** 10.3390/ijerph7010164

**Published:** 2010-01-18

**Authors:** Peter Congdon

**Affiliations:** Department of Geography and Centre for Statistics, Queen Mary University of London, Mile End Rd, London E1 4NS, UK; E-Mail: p.congdon@qmul.ac.uk

**Keywords:** Prevalence, common factor, spatial correlation, coronary heart disease, Bayesian

## Abstract

Different indicators of morbidity for chronic disease may not necessarily be available at a disaggregated spatial scale (e.g., for small areas with populations under 10 thousand). Instead certain indicators may only be available at a more highly aggregated spatial scale; for example, deaths may be recorded for small areas, but disease prevalence only at a considerably higher spatial scale. Nevertheless prevalence estimates at small area level are important for assessing health need. An instance is provided by England where deaths and hospital admissions for coronary heart disease are available for small areas known as wards, but prevalence is only available for relatively large health authority areas. To estimate CHD prevalence at small area level in such a situation, a shared random effect method is proposed that pools information regarding spatial morbidity contrasts over different indicators (deaths, hospitalizations, prevalence). The shared random effect approach also incorporates differences between small areas in known risk factors (e.g., income, ethnic structure). A Poisson-multinomial equivalence may be used to ensure small area prevalence estimates sum to the known higher area total. An illustration is provided by data for London using hospital admissions and CHD deaths at ward level, together with CHD prevalence totals for considerably larger local health authority areas. The shared random effect involved a spatially correlated common factor, that accounts for clustering in latent risk factors, and also provides a summary measure of small area CHD morbidity.

## 1. Background

Profiling geographic variations in health care need is important for equitable and effective targeting of resources that reflects inequalities in morbidity [1]. Coronary heart disease (CHD) is an important part of the overall disease burden faced by government health agencies and demonstrates considerable geographic inequality. Assessing prevalence variations between populations and areas for chronic diseases such as CHD is a central aspect of defining health care need. However, prevalence is not necessarily as well recorded as other health outcomes (e.g., mortality, hospitalisations). For example, in some countries such as the US, area prevalence estimates can only be made on the basis of health survey data. Focussed studies, such as the British Regional Heart Study (BRHS) considered by Morris *et al*. [2] may also provide evidence of geographic prevalence variations, but generally provide only limited geographic coverage; thus the BRHS included only 24 British towns. In recent years, prevalence of major conditions (including CHD) treated in primary care in England has been administratively recorded under a system known as the Quality Outcomes Framework (QOF), but not at a disaggregated spatial scale (e.g., for small areas with populations under 10 thousand). However, small area contrasts are important in defining variations in health need within local health authorities.

While treated prevalence totals are only available for local health agencies (known as Primary Care Trusts or PCTs, with 152 such PCTs in England), deaths and hospital admissions for coronary heart disease are available for smaller areas known as wards, of which there are over 8000. As argued above, estimating CHD prevalence at small area level is important, and this paper develops a shared random effect (or common factor) method to pool information regarding spatial morbidity contrasts over multiple indicators (deaths, hospitalizations, prevalence). This provides a summary index for representing small area CHD morbidity which is applied to estimate small area CHD prevalence totals and hence relative prevalence risks (comparing actual to expected prevalence). Geographic variations in latent constructs relevant to population health are typically spatially correlated, and this is recognised in the derivation of the common morbidity factor [3]. The shared random effect approach also incorporates differences between areas in deprivation levels and other forms of population risk (e.g., ethnic structure). That is, the common factor is partly predicted on the basis of known ecological risk factors or ”multiple causes”, so providing a spatial adaptation of a multiple indicator-multiple cause approach [4].

An illustration is provided by data for London. London may be disaggregated into 625 small areas known as wards, and into 31 Primary Care Trust areas. Some observed data on CHD are at ward level, namely hospital admissions and mortality totals. However, some data (namely CHD prevalence totals) are only observed for considerably larger PCTs. We wish to obtain a summary index of CHD morbidity (as a shared random effect) using all the observations, and use this index to estimate the disaggregated prevalence totals for the 625 wards.

A fully Bayesian approach is used in specifying the model and in the London case study application. This involves ascribing prior densities to model parameters and updating those densities via the likelihood for the observed data. Iterative Monte Carlo Markov Chain (MCMC) techniques [5] are used for estimation, as implemented in the WINBUGS program [6].

## 2. Modelling Latent Morbidity at the Lower Spatial Scale

Let *j* = 1, .., *N_L_* denote the set of lower level small areas within a particular region, and let *i* = 1, .., *N_H_* denote the set of aggregated higher level areas (e.g., local health authorities) within which the small areas are nested. The available data contain *P* observed indicators *y_j_* = (*y_j_*_1_, .., *y_jP_*) at the small area scale (such as small area death totals), and counts *Z_i_* = (*Z_i_*_1_, .., *Z_iQ_*) (e.g., disease prevalence totals) observed only at the aggregated area scale. However, one aim of the modelling process is to develop estimates *z_j_* = (*z_j_*_1_, .., *z_jQ_*) of these indicators at a small area scale.

It is assumed that correlations between the observed indicators can be represented by underlying common latent factors *f* = (*f*_1_, .., *f_R_*), where *R* is of typically of much smaller dimension than the total number *P* + *Q* of observed indicators. For simplicity, a univariate common factor *f* = (*f*_1_*, ...f_N_L__*) is considered (*i.e. R* = 1). In the parlance of factor analysis techniques, the set of observed indicators are proxies for, or ”measures of”, the underlying latent factor.

The first set of small area measurement equations describe the relationship between the observations *y_jp_* (*j* = 1, .., *N_L_*, *p* = 1, …, *P*) and the latent factor. In population health applications, the indicators are typically discrete counts (e.g., deaths, hospital admissions), assumed either Poisson or binomial, so that a general linear mixed model is appropriate for the measurement equations. In the application here, mortality or admission is infrequent in relation to population at risk, and Poisson sampling is relevant. Expected mortality or admission counts *O_jp_* are obtained by applying a standard age-sex schedule (for the entire region, providing an internal standard, or for the nation, providing an external standard) to small area populations at risk. Then one has

(1) $$\begin{array}{l} y_{jp}∼Po(\mu_{jp})\\ \mu_{jp}=O_{jp}\rho_{jp}, \end{array}$$

where *ρ_jp_* is the relative risk of outcome *p* in small area *j*. In the present application, expectations *O_jp_* are scaled to equal the total of expected counts over all small areas, namely $\sum_{j}y_{jp}=\sum_{j}O_{jp}$, so that the region wide average relative risk *ρ_p_* for indicator *p* is 1 if an internal standard is used.

As is conventional for Poisson responses, a log link is employed [7]. So one has measurement models for small area indicators *p* = 1, .., *P,*

(2) $$log(\mu_{jp})=\text{log}(O_{jp})+\lambda_{p}f_{j}+u_{jp},$$

where the unique errors $u_{jp}∼N(0,\sigma_{u}^{2})$ may be necessary for explaining any residual overdispersion. In substantive terms, the *u_jp_* also control for structural influences unrelated to population morbidity per se (e.g., effectiveness of health care services, hospital configuration). Intercepts are not included in (2)*,* so providing a form of location constraint on the latent variable *f* [8]. The coefficients *λ_p_* are typically known as loadings, the specification of which is considered below.

Variations in population morbidity, whether observed or latent, typically display spatial correlation between adjacent areas-unmeasured aspects of population structure relevant to health risk typically straddle administrative boundaries [9]. However, rather than a priori assume exclusively spatial dependence, the model here determines an appropriate mix between spatial and non-spatial (”exchangeable”) dependence in the latent morbidity construct.

There may also be observed variables (*i.e.*, known rather than latent risk factors) that are relevant to defining the common morbidity factor. For example, many indices of health need are composites of variables such as unemployment rates, poverty rates, car ownership, *etc.* Here a spatial adaptation of the multiple indicators-multiple causes (MIMIC) approach is used, with *L* measured causes *x_j_* = (*x_j_*_1_, .., *x_jL_*)*′* (such as small area socio-economic or population risk variables) of the latent morbidity index. These influence the latent morbidity index *f_j_* via regression terms

(3.1) $$\eta_{j}=\beta x_{j}=\beta_{1}x_{j1}+\ldots +\beta_{L}x_{jL}$$

where the regression excludes an intercept, with residuals denoted

(3.2) $$r_{j}=f_{j}-\eta_{j}.$$

Additionally if the *x_jl_* are standardised, the absolute size of the *β* coefficients measures the relative importance of different population risk factors or socio-economic variables in defining the morbidity index.

To allow a mix between spatial and non-spatial dependence in the latent index, define a spatial correlation parameter *k* ∈ (0, 1), and assume symmetric spatial interactions *w_jh_*. Also let *f*_[_*_j_*_]_ = (*f*_1_*,* …*f_j_*_−1_, *f_j_*_+1_, …, *f_N_L__*) denote the collection of morbidity effects for all areas but area *j.* Under the scheme of Leroux *et al.* [10], though adapted here to include regression effects, as in (3.1) (3.2), the expected value of the latent effect in area *j* and its variance are

(4.1) $$E(f_{j}|f_{\left[j\right]})=\eta_{j}+\kappa \sum_{h\neq j}w_{jh}r_{h}/[1-\kappa +\kappa \sum_{h\neq j}w_{jh}],$$

(4.2) $$Var(f_{j}|f_{\left[j\right]})=\frac{\sigma_{f}^{2}}{\left[1-\kappa +\kappa \sum_{h\neq j}w_{jh}\right]},$$

where $\sigma_{f}^{2}$ is a variance parameter. A value of *k* close to 1 indicates high spatial dependence in latent morbidity, while values near zero imply lack of spatial correlation.

The *w_jh_* may incorporate factors such as distances between areas *j* and *h*. However, in many applications the *w_jh_* simply represent adjacency, namely *w_jh_* = *w_hj_* = 1 if areas *h* and *j* are adjacent, zero otherwise. In this case it is relevant to define the neighbourhood *∂_j_* of small area *j*, which contains the *m_j_* areas adjacent to area *j*, and one then has $\sum_{h\neq j}w_{jh}=m_{j}$. The expectations are then

(5) $$E(f_{j}|f_{\left[j\right]})=\eta_{j}+\kappa \sum_{h\in \partial_{j}}r_{h}/[1-\kappa +\kappa m_{j}].$$

To uniquely determine the scale of the *f* scores, constraints are needed on the loadings *λ_p_*, or on the variance $\sigma_{f}^{2}$ in (4.2). The first kind involves standardized factors, with $\sigma_{f}^{2}=1$, as in the spatial factor model of Wang and Wall [11], with all loadings then unknowns. An alternative constraint involves appropriately fixed loadings, such as setting one of the loadings *λ_p_* to a particular fixed value, usually 1. The variance $\sigma_{f}^{2}$ is then an unknown parameter.

## 3. Methods: Estimating Prevalence at Small Area Level based on the Morbidity Index

We wish not just to obtain a latent morbidity index, but to use this index to estimate unknown indicator totals (*z_j_*_1_, …, *z_jQ_*) (e.g., prevalence totals) for small areas *j* = 1, …, *N_L_*. Estimation of the missing lower area scale data takes account (a) of values of the small area morbidity index *f* = (*f*_1_, …, *f_N_L__),* and (b) of the known prevalence totals (*Z_i_*_1_, …, *Z_iQ_*) for the *i* = 1, …, *N_H_* higher level areas. The small areas are nested within the higher level areas, with *H_j_* ∈ {1, *…*,*N_H_*} denoting the higher level area to which small areas *j* belong, and the region is defined equivalently by all the higher level areas or all the lower level areas.

Also assumed known are age-sex structures for the small area populations, and from these can be obtained expected totals *E_jq_* of the small area counts *z_jq_*. This involves using an external schedule of prevalence rates *r_qsk_* for the *q^th^* outcome by age *k* and sex *s*, and applying this schedule to small area population estimates *P_jsk_,* so that $E_{jq}=\sum_{s}\sum_{\kappa }P_{js\kappa }r_{qs\kappa }$.

To ensure the estimates of (*z_j_*_1_,…, *z_jQ_*) take account of the observed prevalence counts *Z_iq_* of the higher level areas they are located in, the Poisson means *Δ_iq_* in the likelihood *Z_iq_* ∼ *Po*(*Δ_iq_*) for the higher level observed totals *Z_iq_* are defined by totals of small area means *δ_jq_* located within each higher area. Thus let

(6) $$\Delta_{iq}=\sum_{H_{j}=i}\delta_{jq},$$

denote the total mean prevalence counts for large areas *i* obtained from the small area model for the *z*-indicators.

The small area model (*i.e.*, the model for the *δ_jq_*) can be set up to ensure that the posterior means of the *Δ_iq_* equal (to a close approximation) the known higher level totals *Z_iq_*. One way to achieve thus is via a collection of *N_H_* fixed effects *γ_q_,H_j_* in the model for the *δ_jq_,* equivalent to using dummy variables in the small area model for each higher scale area, and providing a Poisson equivalence to the multinomial [12]. Thus the *z_jq_* for *H_j_* = *i* are multinomial within *Z_iq_*. We also wish the values of the latent morbidity index *f_j_* to influence the multinomial allocation of *Z_iq_* to small areas in a manner analogous to that in Equation (2). So the small area model is

(7.1) $$z_{jq}∼Po(\delta_{jq}),$$

(7.2) $$log(\delta_{jq})=\text{log}(E_{jq})+\gamma_{q,H_{j}}+\lambda_{P+q}f_{j},$$

where *λ_P_*_+_*_q_* are additional loadings on the latent spatial morbidity index. Whether they are set to known values or taken as unknowns depends on the identification constraint adopted for the scale of the *f_j_*.

Other priors for (*γ*1, …,*γ_N_H__*), for example, as random rather than fixed effects, in practice have a very similar consequence. that the means of the *Δ_iq_* equal (to a close approximation) the known higher level total *Z_iq_*. For example, one might use random effect spatial priors, comparable to (4)–(5) but at the higher area level.

In some circumstances, there may be doubts about how far the *Z_iq_* are accurate measures of morbidity, and a constraint to reproduce them may not be advantageous. For example, the prevalence counts obtained under the QOF system in England may under-record prevalence in deprived areas, since the quality of primary care is lower in such areas [13], this may result in less effective case-finding [14]. To allow unconstrained estimation of small area prevalence counts, one may use an intercept in the model for *δ_jq_* that is not specific to the higher area, namely

(8) $$log(\delta_{jq})=\text{log}(E_{jq})+\gamma_{q}+\lambda_{P+q}f_{j}.$$

This model ensures $\sum_{i=1}^{N_{H}}Z_{iq}=\sum_{j=1}^{N_{L}}z_{jq}$, but does not guarantee that $\sum_{H_{j}=i}z_{jq}=Z_{iq}$, as the constrained model does.

To recap, the model is a form of spatial structural equation model (SEM) that seeks to estimate small area health outcomes *z* for which only large area observations *Z* are available. The model works in practice by using observed small area health indicators *y* (e.g., mortality from a particular disease) which are likely to be correlated with the missing small area outcomes *z* (e.g., prevalence of the same disease). The information in the correlated multiple indicators *y* is summarised in a latent variable *f* that depends on observed area risk factors *x*, but is also spatially correlated, reflecting spatial clustering in unobserved area risk factors. The decomposition of large area totals *Z* to small areas is based on the latent variable *f,* and the decomposition can be constrained so that total small area prevalences *z_j_* sum to the known *Z_i_* for large area *i* within which areas *j* are located. It seems reasonable to use socioeconomic variables *x* as causes of variability in *f*, but another strategy would be to use small area socioeconomic variables as additional indicators of the latent variable.

## 4. CHD Morbidity in London: Data

The motivating case study illustrating the above methodology involves derivation of a univariate index of CHD morbidity (*i.e. R* = 1) for London small areas using *P* = 4 observed small area health indicators, and a single health indicator (*Q* = 1) observed only at an aggregated area scale. The two area scales are wards and Primary Care Trusts (PCTs): there are *N_L_* = 625 wards and *N_H_* = 31 PCTs in London. The first two small area indicators (*y_j_*_1_*, y_j_*_2_) are male and female CHD deaths over 2004–2006, while (*y_j_*_3_*, y_j_*_4_) are male and female hospitalisations for CHD over three financial years 2003–2004 to 2005–2006. Expected deaths and hospitalisations *O_jp_* in (2) are based on London wide death and hospitalisation rates specific to gender and five year age bands.

CHD prevalence totals *Z_i_* (for 2004–2005 and 2005–2006 combined) are observed only for PCTs, but one goal of the model is to estimate missing small area CHD prevalence totals *z_j_*. Expected CHD prevalence totals *E_jq_* = *E_j_* at ward level in (7.2) are obtained with an external schedule of CHD prevalence rates by age and sex, and applying this schedule to small area population estimates (here 2005 intercensal estimates of ward populations developed by the UK Office of National Statistics). The external schedule used is based on the 2003 Health Survey for England [15], with the expectations *E_j_* scaled so that the London wide standard prevalence ratio is 1 (*i.e.*, the total of observed prevalence counts *Z_i_* across all London PCTs equals the total of expected prevalence counts *E_j_* over all London wards).

In the multiple cause sub-model (3), there are *L* = 3 socio-economic indicators of population CHD risk. These are *x*_1_ = average weekly household income in 2001–2002 [16], *x*_2_ = proportion of population of south Asian ethnicity, 2001 Census [17,18], and *x*_3_ = estimated ward level smoking prevalences [19]. These predictors are converted to standardised form so that their relative importance can be assessed.

## 5. CHD Morbidity in London: Models

Two models are compared. One assumes intercepts in the small area prevalence model that vary by PCT, as in (7.2). The other is unconstrained, as in (8). Identifiability is achieved by setting *λ_P_*_+1_ = *λ*_5_ = 1, so that $\sigma_{f}^{2}$ is an unknown, the inverse variance $1/\sigma_{f}^{2}$ is accordingly assigned a Gamma(1,1) prior. To ensure the model produces a positive index of CHD morbidity, the remaining *λ_p_* parameters also follow Gamma(1,1) priors [20]. Fixed effect parameters, namely *β* parameters in (3) and *γ* parameters in (7.2) and (8) are assigned diffuse *N*(0, 100) priors, while a uniform prior *k* ∼ *U*(0, 1) is assumed for the spatial correlation coefficient in (4)–(5).

Comparisons of model fit use the deviance information criterion (*DIC*) [21], obtained as the average deviance plus a complexity measure. The focus is on goodness of fit for the y-indicators (deaths and hospital admissions). Model 1 will automatically fit the higher level (PCT) prevalence data better as it has separate intercepts for each PCT. Model checking is based on the posterior predictive density, *p*(*y_rep_*|*y*)*,* under a mixed predictive approach [22], where sampled replicates *y_rep_* are based on model means that include replicate samples from random effects (*f* and *u* effects). Then a mixed-predictive test for area *j* and outcome *p* has the form

$$p_{jp,mix}=Pr(y_{rep,jp}>y_{jp}|y)+0.5Pr(y_{rep,jp}=y_{jp}|y),$$

with extreme tail values indicating poorly fitted cases. One may compare the proportion of cases under-fitted (*p_jp,mix_ <* 0:05) or over-fitted (*p_jp,mix_ >* 0:95) with the expected proportions in these two tails (namely 0.05 in each).

Inferences are based on the second halves of two chain runs of 10000 iterations with convergence before iteration 5000 assessed using Gelman-Rubin scale reduction factors [23]. Table 1 presents model fit and checking criteria. It can be seen that model 1 (ward totals constrained to reproduce the QOF totals at PCT level) has a lower DIC, and satisfactory predictive performance. Table 2 summarises parameter estimates under the two models.

The estimated *k* coefficients in Table 2 indicate a high spatial correlation in the latent CHD morbidity index under both models. The estimated *β_l_* parameters from the multiple cause regression (3) show income differences between wards to be the most important known influence on the index, though concentrations of south Asian ethnic groups are also important. As expected, higher income levels are negatively associated with morbidity (so the 95% interval for the coefficient *β*_1_ is confined to negative values). The importance of area socioeconomic status to CHD outcomes is confirmed by other studies [24,25].

The income effect is weaker in the constrained model. This is likely to reflect discrepancies in some deprived parts of London between officially recorded prevalence (used as a constraint in model 1), and what would be expected on the basis of socioeconomic structure. Examples are the apparently low prevalence in some deprived areas in inner South East London. The consequence is that the effect of income is deflated, providing an example of measurement error affecting regression estimates. Table 3 compares prevalence (in the higher level PCT areas) based on the official QOF totals, with average income levels in such areas (weekly income in hundreds of pounds). Outliers in the negative relation between prevalence and income (there is a-0.50 correlation between PCT ranks for prevalence risk and for income, even though using official CHD prevalence data) include the deprived inner SE London area of Southwark. The latter area has the sixth lowest income, but also low measured prevalence. So while the DIC criterion prefers model 1, the geographic prevalence pattern implied by the unconstrained model 2 might be preferred on the basis of epidemiological arguments.

**Figure 1.**
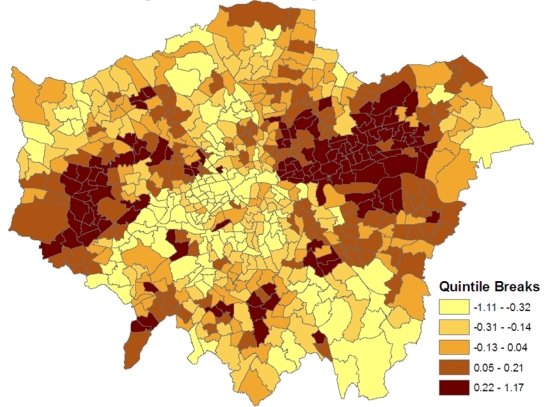
Latent Morbidity Index.

**Figure 2.**
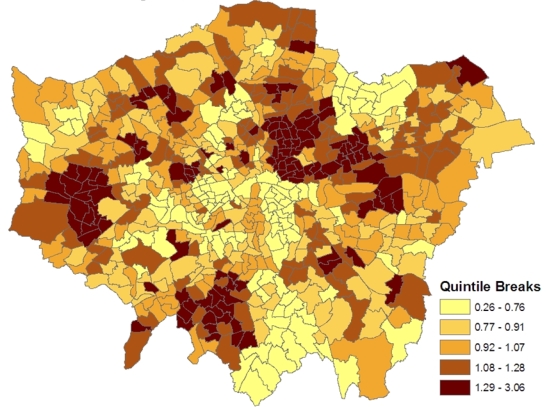
Relative prevalence risk.

**Figure 3.**
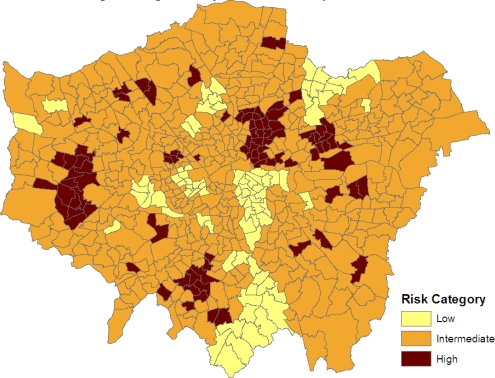
Significantly elevated and depressed risk.

Figure 1 shows the spatial patterning of the CHD morbidity scores in model 1, higher values are in inner east (though not central) London and in certain parts of west London. Figure 2 maps the estimated ward level prevalences in terms of relative risks under the constrained model 1, namely the posterior means of *ξ_j_* = *z_j_*/*E_j_*. For policy purposes, the probability that a small area has significantly higher relative risk and thus possibly needs special resources is important. Therefore the marginal variance *ω*^2^ = *var*(*ξ_j_*) is monitored during the MCMC run, and the standardized relative risks (SRRs)

$$SRR_{j}=(\xi_{j}-\bar{\xi })/\omega$$

are also monitored. High posterior means for these SRRs (e.g., SRRs above 1 or 2, namely 1 or 2 standard deviations above average) indicate significantly elevated prevalence, while low values (under −1, or under −2) indicate significantly low prevalence. Figure 3 maps the three categories: *SRR_j_ >* 1, −1 *< SRR_j_ <* 1 and *SRR_j_ <* −1. Clusters of elevated risk are now clearly apparent.

## 6. Discussion

Estimates of prevalence at small area level are often necessary, as prevalence is less likely to be routinely reported for such areas, whereas outcomes such as mortality and hospitalization often are. Prevalence totals may, however, be reported for relatively aggregated large areas, either from health surveys, or (in the case of the UK) systems of chronic disease monitoring in primary care. The present paper has employed a common spatial factor model to disaggregate large area CHD prevalence totals to small areas. Various forms of common spatial factor model have been proposed for spatial health outcomes epidemiology. For example, Hogan & Tchernis [26] develop a small area measurement model for a socioeconomic deprivation score, while Liu *et al.* [27] develop a spatial structural equation model linking health outcomes to spatially correlated latent indices.

The present paper includes three main extensions on such work: first, it allows for known risk factors to influence the composite index via a multiple cause sub-model, second, it allows the data to determine the extent of spatial correlation rather than presuming a priori that latent factors are necessarily spatially dependent, and third, it applies the model to estimate missing health outcomes at a lower area scale (CHD prevalence for wards in the London case study), when observations on such outcomes are only available at a higher scale (PCTs in the London study). The essence of the method is to use all available lower scale information (both from levels of related health outcomes *y* and from measures of socioeconomic structure *x*) to provide a reasonable imputation of the missing outcomes *z* at the lower scale.

The case study has considered deaths and hospital admissions for CHD as the lower scale observed data (the *y*-variables), and a single higher scale outcome (CHD prevalence), with *x*-variables (causes) being income, ethnicity and smoking. It has also had a primarily urban focus, being confined to London. Under a broader geographic focus (including rural small areas), it might be relevant to consider adding an urban-rural indicator to the *x*-variables.

As demonstrated in Figure 3, one application of the modelling scheme is to highlight small areas with significantly elevated prevalence. This is important for prioritizing resourcing and intervention, and is based on a method that seeks to make use of all relevant information (comorbid outcomes, area social structure, and the spatial configuration of small areas). By contrast, many other health needs measures used to distribute resources are based simply on socioeconomic variables (e.g., the Jarman score) [28], or on regressions of single health indicators (e.g., hospitalizations) on socioeconomic variables [29], when multiple indicators may in fact be relevant. Existing methods also neglect spatial clustering in unobserved risk factors.

The methodology set out here has potential application to small area prevalence estimation for other chronic diseases, though the appropriate mix of *y* and *x*-variables would be different. For example, the Quality Outcomes Framework system in the UK monitors prevalence of several chronic diseases. In particular, PCT (higher scale) level counts of the prevalence of serious mental illness (SMI) are available, but one may seek ward level measures of SMI prevalence. The available *y*-indicators in this situation might be small area hospital admissions for conditions such as schizophrenia and bipolar disorder, while *x*-variables would include indicators of risk for psychiatric morbidity, such as small area income or deprivation, urban-rural status, social capital and so on.

Another potential application area is to use health survey information on disease prevalence, often obtained only for higher scale regional units. For example, the public release version of recent Health Surveys for England only contains prevalence rate estimates for chronic conditions included in the survey (e.g., obesity, diabetes) for 10 Strategic Health Authorities. However, one may wish to use this information in making estimates of such conditions for lower scale geographies such as the 354 local authorities in England. Using survey based regional estimates *Z_i_* of prevalence, one can estimate lower scale totals *z_j_*, using information on both socioeconomic structure (*x_j_*) and related outcomes (*y_j_*) at the lower spatial scale. The procedures outlined in the paper could in fact be used to disaggregate survey based estimates *Z_ik_* which include relevant demographic stratifiers *k* (e.g. age, sex, ethnicity). Relevant spatial SEM coefficients (*β* and *λ* parameters) may well differ between demographic category. For example, one might seek to disaggregate survey-based regional estimates of diabetes by ethnicity to a lower spatial scale.

## Figures and Tables

**Table 1.** Fit measures.

	Average Deviance	Complexity	DIC	Proportion of y values with Pr(y*_rep_* > *y*) under 0.05 or over 0.95
Model 1 (Multinomial Constraint)	2,570	1,290	3,860	0.093
Model 2 (unconstrained)	2,529	1,512	4,041	0.085

**Table 2.** Summary of model parameter estimates.

		Mean	Stdevn	Monte Carlo SE	2.5%	97.5%
Model 1	*β*_1_	−0.199	0.020	0.002	−0.242	−0.162
	*β*_2_	0.180	0.023	0.002	0.139	0.231
	*β*_3_	0.050	0.020	0.002	0.009	0.087
	*κ*	0.937	0.055	0.002	0.800	0.998
	*λ*_1_	0.450	0.039	0.003	0.371	0.525
	*λ*_2_	0.410	0.044	0.003	0.325	0.495
	*λ*_3_	0.720	0.040	0.004	0.638	0.783
	*λ*_4_	0.769	0.041	0.004	0.682	0.893
Model 2	*β*_1_	−0.394	0.032	0.003	−0.461	−0.333
	*β*_2_	0.158	0.025	0.0001	0.108	0.208
	*β*_3_	0.062	0.030	0.002	0.007	0.120
	*κ*	0.935	0.055	0.002	0.797	0.998
	*λ*_1_	0.303	0.024	0.001	0.258	0.352
	*λ*_2_	0.241	0.026	0.001	0.191	0.292
	*λ*_3_	0.366	0.020	0.001	0.326	0.406
	*λ*_4_	0.413	0.023	0.001	0.366	0.456

**Table 3.** PCT prevalence risks based on official prevalence total, compared with income patterns.

PCT	Observed from QOF	Expected using HSE 2003 as standard	RR based on actual QOF prevalence records	Rank of RR	Average income	Income rank
Barking and Dagenham	9,800	9,147	1.071	25	5.3	2
Barnet	20,161	19,287	1.045	21	7.6	25
Bexley	13,973	14,778	0.946	12	6.7	14
Brent	14,040	13,542	1.037	20	6.6	10
Bromley	19,466	20,883	0.932	11	7.5	23
Camden	9,430	9,389	1.004	15	7.3	21
City and Hackney	9,030	8,746	1.033	19	5.5	4
Croydon	17,519	18,868	0.928	9	6.8	15
Ealing	18,410	15,228	1.209	29	7.3	22
Enfield	14,839	16,232	0.914	5	6.6	11
Greenwich	12,419	11,464	1.083	26	5.8	5
Hammersmith and Fulham	7,022	7,609	0.923	7	7.8	27
Haringey	9,318	9,360	0.996	14	6.6	12
Harrow	13,680	12,949	1.056	22	7.7	26
Havering	16,650	16,538	1.007	16	6.9	16
Hillingdon	13,929	14,408	0.967	13	7.2	19
Hounslow	8,127	7,663	1.061	24	6.5	9
Islington	13,929	14,408	0.967	13	7.2	19
Kensington and Chelsea	6,953	9,506	0.731	1	8.0	28
Kingston	8,573	8,485	1.010	17	8.1	29
Lambeth	9,768	10,499	0.930	10	6.6	13
Lewisham	12,027	11,348	1.060	23	6.1	8
Newham	12,495	9,433	1.325	31	4.8	1
Redbridge	14,488	14,223	1.019	18	6.9	17
Richmond and Twickenham	7,802	10,312	0.757	2	9.0	31
Southwark	10,233	11,168	0.916	6	6.0	6
Sutton and Merton	19,303	21,385	0.903	4	7.6	24
Tower Hamlets	9,724	7,523	1.293	30	5.4	3
Waltham Forest	11,955	10,640	1.124	27	6.0	7
Wandsworth	10,904	11,763	0.927	8	8.4	30
Westminster	9,921	11,097	0.894	3	7.1	18
